# Supplementary material for: Brief Hospital Supervision of Exercise and Diet During Adjuvant Breast Cancer Therapy Is Not Enough to Relieve Fatigue: A Multicenter Randomized Controlled Trial
Source: Nutrients. 2020 Oct 9;12(10):3081. doi: 10.3390/nu12103081 (PMC7600233; doi:10.3390/nu12103081)
Supplement: Supplementary file 1 [file nutrients-12-03081-s001.zip › SFig5.DOCX]

**Figure S5.** Evolution of the different physical activity domains of the GPAQ questionnaire in the intention-to-treat population. Data are presented as mean+SD.

| **Total MET (MET·min/wk)** |
| --- |
|  |
| **Recreational activity - moderate intensity (MET·min/wk)** |
|  |

| **Recreational - vigorous intensity (MET·min/wk)** |
| --- |
|  |
| **Work - moderate intensity (MET·min/wk)** |
|  |
| **Work - vigorous intensity (MET·min/wk)** |
|  |

| **Travel - moderate intensity (MET·min/wk)** |
| --- |
|  |
| **Sedentary time (minutes/day) by randomization arm** |
|  |
