# Supplementary material for: Brief Hospital Supervision of Exercise and Diet During Adjuvant Breast Cancer Therapy Is Not Enough to Relieve Fatigue: A Multicenter Randomized Controlled Trial
Source: Nutrients. 2020 Oct 9;12(10):3081. doi: 10.3390/nu12103081 (PMC7600233; doi:10.3390/nu12103081)
Supplement: Supplementary file 1 [file nutrients-12-03081-s001.zip › SFig3-4.DOCX]

**Figure S3.** Evolution of quality of life (EORTC QLQ-C30) in the intention-to-treat population. Data are presented as mean+SD.

| **Global health status** |
| --- |
|  |
| **Physical functioning** |
|  |
| **Role functioning** |
|  |

| **Emotional functioning** |
| --- |
|  |
| **Cognitive functioning** |
|  |
| **Social functioning** |
|  |
| **Symptom: Fatigue** |
|  |

**Figure S4.** Evolution of quality of life (EORTC QLQ-C30) in the per protocol population. Data are presented as mean+SD.

| **Global health status, PP** |
| --- |
|  |
| **Physical functioning, PP** |
|  |
| **Role functioning, PP** |
|  |

| **Emotional functioning, PP** |
| --- |
|  |
| **Cognitive functioning, PP** |
|  |
| **Social functioning, PP** |
|  |

| **Symptom: Fatigue, PP** |
| --- |
|  |
