# Supplementary material for: Brief Hospital Supervision of Exercise and Diet During Adjuvant Breast Cancer Therapy Is Not Enough to Relieve Fatigue: A Multicenter Randomized Controlled Trial
Source: Nutrients. 2020 Oct 9;12(10):3081. doi: 10.3390/nu12103081 (PMC7600233; doi:10.3390/nu12103081)
Supplement: Supplementary file 1 [file nutrients-12-03081-s001.zip › SFig1-2.docx]

**Figure S1.** General Fatigue subscale of the MFI20 according to EPICE strata (precariousness level). Data are presented as mean+SD.

|  |
| --- |

**Figure S2.** General Fatigue subscale of the MFI20 according to randomization arms stratified by EPICE score (precariousness level). Data are presented as mean+SD. A) General fatigue in patients with EPICES scores of 0.0 - 16.56 (non-precarious population, N=218). APAD effect: -0.017 [95% CI -0.11; 0.077]; p=0.724. B) General fatigue in patients with EPICE scores of 16.56 - 48.52 (intermediate, N=120). APAD effect vs. Control: 0.12 [-0.021; 0.25]; p=0.098.C) General fatigue in patients with EPICE scores of 48.52 - 100 (precarious population, N=22). APAD effect: 0.10 [-0.15; 0.36]; p=0.438.

| **A.** |
| --- |
|  |
| **B.** |
|  |
| **C.** |
|  |
