# Supplementary material for: Brief Hospital Supervision of Exercise and Diet During Adjuvant Breast Cancer Therapy Is Not Enough to Relieve Fatigue: A Multicenter Randomized Controlled Trial
Source: Nutrients. 2020 Oct 9;12(10):3081. doi: 10.3390/nu12103081 (PMC7600233; doi:10.3390/nu12103081)
Supplement: Supplementary file 1 [file nutrients-12-03081-s001.zip › STab1-2.docx]

**Table S1.** Fatigue sub-scales of the MFI20 over time in the per protocol population.

|  |  | | Baseline (T0) | | | | End of CT (T1) | | | | | End of RT (T2) | | | | 1 year after inclusion (T3) | | | | LMM coefficients^1^ [95% CI] |
| --- | --- | --- | --- | --- | --- | --- | --- | --- | --- | --- | --- | --- | --- | --- | --- | --- | --- | --- | --- | --- |
|  |  | | Mean | | SD | | Mean | | SD | | p | Mean | | SD | p | Mean | | SD | p |  |
| General fatigue (Endpoint) | Control | | 9.62 | | 4.19 | | 12.19 | | 4.21 | | 0.471 | 10.79 | | 4.44 | 0.158 | 10.58 | | 3.84 | 0.255 | β_1_=0.20 [-0.58; 0.97], p=0.622 |
|  | APAD | | 9.78 | | 4.09 | | 11.80 | | 4.26 | |  | 11.55 | | 4.50 |  | 11.09 | | 3.96 |  |  |
| *Median (range) of the RD ^2^* | | Control | |  | |  | 0.25 (-0.67; 3.0) | | | | | 0.083 (-0.60; 3.50) | | | | 0.20 (-0.69; 3.5) | | | |  |
|  | | APAD | |  | |  | 0.20 (-0.5; 2.75) | | | | | 0.18 (-0.58; 2.25) | | | | 0.15 (-0.6; 3.0) | | | |  |
|  | |  | |  | |  | p=0.141 | | | | | p=0.297 | | | | p=0.983 | | | |  |
|  | |  | |  | |  |  |  | |  | |  |  | |  |  |  | |  |  |
| Physical fatigue | | Control | | 9.54 | | 3.71 | 11.28 | 4.05 | | 0.961 | | 10.61 | 3.96 | | 0.833 | 10.10 | 3.63 | | 0.504 | β_1_=-0.26 [-0.97; 0.45], p=0.469 |
|  | | APAD | | 8.94 | | 3.75 | 11.25 | 4.18 | |  | | 10.56 | 4.34 | |  | 9.92 | 3.83 | |  |  |
| Mental fatigue^3^ | | Control | | 7.66 | | 3.80 | 9.19 | 4.26 | | 0.866 | | 8.90 | 4.31 | | 0.769 | 8.82 | 4.26 | | 0.910 | β_2_=-0.020 [-0.04; 0.002], p=0.069 |
|  | | APAD | | 8.49 | | 4.00 | 9.04 | 4.15 | |  | | 8.87 | 4.57 | |  | 8.80 | 4.32 | |  |  |
| Reduced activities | | Control | | 8.33 | | 3.63 | 10.12 | 4.26 | | 0.407 | | 9.14 | 3.92 | | 0.684 | 8.75 | 4.02 | | 0.994 | β_1_=0.06 [-0.68; 0.79], p=0.882 |
|  | | APAD | | 8.74 | | 3.90 | 9.76 | 4.59 | |  | | 9.46 | 4.42 | |  | 8.66 | 3.76 | |  |  |
| Reduced motivation^3^ | | Control | | 7.72 | | 3.49 | 8.22 | 3.92 | | 0.691 | | 7.84 | 3.46 | | 0.863 | 8.07 | 3.19 | | 0.275 | β_2_=-0.023 [-0.39; -0.008], p=0.003 |
|  | | APAD | | 8.42 | | 3.52 | 8.15 | 3.45 | |  | | 7.94 | 3.51 | |  | 7.74 | 3.42 | |  |  |
| **EORTC QLQ-C30** | |  | |  | |  |  |  | |  | |  |  | |  |  |  | |  |  |
| Global health status | | Control | | 69.94 | | 18.67 | 59.39 | 21.22 | | 0.396 | | 66.55 | 19.53 | | 0.383 | 67.44 | 19.30 | | 0.250 | β_1_=0.017 [-.052; 0.088], p=0.624 |
|  | | APAD | | 69.31 | | 18.92 | 61.64 | 20.43 | |  | | 64.6 | 19.19 | |  | 71.18 | 15.28 | |  |  |
| Physical functioning | | Control | | 87.17 | | 14.59 | 79.79 | 19.10 | | 0.250 | | 84.40 | 15.38 | | 0.043 | 85.45 | 17.26 | | 0.149 | β_1_=0.025 [-.034; 0.084], p=0.800 |
|  | | APAD | | 89.53 | | 13.27 | 81.88 | 19.80 | |  | | 86.76 | 17.65 | |  | 89.88 | 1268 | |  |  |
| Role functioning | | Control | | 84.46 | | 22.16 | 77.99 | 23.34 | | 0.237 | | 84.35 | 21.03 | | 0.782 | 87.05 | 18.48 | | 0.349 | β_1_=0.055 [-.045; 0.16], p=0.283 |
|  | | APAD | | 88.38 | | 18.08 | 80.62 | 23.83 | |  | | 83.86 | 22.80 | |  | 90.43 | 1515 | |  |  |
| Emotional functioning | | Control | | 63.70 | | 23.52 | 72.75 | 24.83 | | 0.443 | | 75.51 | 23.09 | | 0.489 | 73.26 | 20.10 | | 0.272 | β_1_=0.080 [-0.03; 0.19], p= 0.153 |
|  | | APAD | | 65.92 | | 21.41 | 72.33 | 20.87 | |  | | 74.45 | 21.84 | |  | 76.03 | 19.90 | |  |  |
| Cognitive functioning | | Control | | 85.21 | | 20.50 | 79.30 | 25.14 | | 0.800 | | 80.16 | 21.75 | | 0.926 | 79.97 | 23.42 | | 0.884 | β_1_=4.1e-06 [-0.096; 0.096], p=1.000 |
|  | | APAD | | 84.98 | | 19.07 | 79.52 | 23.35 | |  | | 79.79 | 22.48 | |  | 81.48 | 21.42 | |  |  |
| Social functioning | | Control | | 82.58 | | 24.15 | 66.77 | 30.63 | | 0.768 | | 73.58 | 27.71 | | 0.488 | 82.04 | 23.99 | | 0.480 | β_1_=0.031 [-0.11; 0.17], p=0.659 |
|  | | APAD | | 85.09 | | 21.72 | 68.45 | 28.74 | |  | | 71.26 | 28.54 | |  | 85.96 | 18.81 | |  |  |
| Fatigue | | Control | | 28.54 | | 21.86 | 44.87 | 27.24 | | 0.970 | | 34.62 | 25.73 | | 0.170 | 32.26 | 21.96 | | 1.000 | β_1_=-0.046 [-0.262; 0.171], p=0.678 |
|  | | APAD | | 27.31 | | 22.67 | 44.96 | 29.43 | |  | | 37.53 | 24.16 | |  | 31.48 | 20.97 | |  |  |

^1^ In the linear mixed model (LMM): β1 is the coefficient of the variable ‘arm’ (interpreted as APAD effect with respect to Control) noted as β_2_ when interaction arm*time was significant, β0 is the coefficient of the variable time.

^2^ For each patient, the relative difference (RD) with respect to the baseline value of the General fatigue subscale at the end of chemotherapy (CT), end of radiotherapy (RT; end of the oncological treatment), and 1 year after the inclusion was calculated as (GFS_endRT_ - GFS_Inclusion_)/ GFS _Inclusion_). A smaller RD indicates a greater reduction in general fatigue.

^3^ A baseline imbalance between arms was observed in the per-protocol population for Mental fatigue (p=0.033) and Reduced motivation (p=0.041).

**Table S2.** Anxiety and depression disorders in the per protocol population.

|  | Control | | APAD | |  |
| --- | --- | --- | --- | --- | --- |
|  | N | % | N | % | p |
| Baseline (T0) |  |  |  |  |  |
| Anxiety |  |  |  |  | 0.820 |
| Absence (<7) | 0 | 0.00 | 0 | 0.00 |  |
| Suspected (8-10) | 2 | 1.12 | 2 | 1.41 |  |
| Confirmed (>10) | 176 | 98.88 | 140 | 98.59 |  |
| Depression |  |  |  |  | 0.264 |
| Absence (<7) | 0 |  | 2 | 1.41 |  |
| Suspected (8-10) | 70 | 39.33 | 58 | 40.85 |  |
| Confirmed (>10) | 108 | 60.67 | 82 | 57.75 |  |
| End of chemotherapy (T1) |  |  |  |  |  |
| Anxiety |  |  |  |  | 0.898 |
| Absence (<7) | 0 | 0.00 | 0 | 0.00 |  |
| Suspected (8-10) | 1 | 0.64 | 1 | 0.76 |  |
| Confirmed (>10) | 156 | 99.36 | 130 | 99.24 |  |
| Depression |  |  |  |  | 0.138 |
| Absence (<7) | 0 | 0.00 | 3 | 2.29 |  |
| Suspected (8-10) | 55 | 35.03 | 49 | 37.40 |  |
| Confirmed (>10) | 102 | 64.97 | 79 | 60.31 |  |
| End of radiotherapy (T2) |  |  |  |  |  |
| Anxiety |  |  |  |  |  |
| Absence (<7) | 0 | 0.00 | 0 | 0.00 |  |
| Suspected (8-10) | 0 | 0.00 | 0 | 0.00 |  |
| Confirmed (>10) | 147 | 100.00 | 127 | 100.00 |  |
| Depression |  |  |  |  | 0.676 |
| Absence (<7) | 1 | 0.68 | 1 | 0.79 |  |
| Suspected (8-10) | 68 | 46.26 | 52 | 40.94 |  |
| Confirmed (>10) | 78 | 53.06 | 74 | 58.27 |  |
| 1 year after inclusion (T3) | |  |  |  |  |
| Anxiety |  |  |  |  | 0.362 |
| Absence (<7) | 0 | 0.00 | 1 | 0.93 |  |
| Suspected (8-10) | 1 | 0.77 | 0 |  |  |
| Confirmed (>10) | 129 | 99.23 | 107 | 99.07 |  |
| Depression |  |  |  |  | 0.026 |
| Absence (<7) | 0 | 0.00 | 2 | 1.85 |  |
| Suspected (8-10) | 43 | 33.08 | 50 | 46.30 |  |
| Confirmed (>10) | 87 | 66.92 | 56 | 51.85 |  |
