# Supplementary material for: Brief Hospital Supervision of Exercise and Diet During Adjuvant Breast Cancer Therapy Is Not Enough to Relieve Fatigue: A Multicenter Randomized Controlled Trial
Source: Nutrients. 2020 Oct 9;12(10):3081. doi: 10.3390/nu12103081 (PMC7600233; doi:10.3390/nu12103081)
Supplement: Supplementary file 1 [file nutrients-12-03081-s001.zip › SFig6.docx]

**Figure S6.** Evolution of nutrient intake and weight control variables (weight, BMI, waist size) by randomization arm in the intention-to-treat population. Data are presented as mean+SD.

| **Total energy (Kcal)** |
| --- |
|  |
| **Animal proteins (g)** |
|  |
| **Vegetal proteins** |
|  |

| **Lipids (g)** |
| --- |
|  |
| **Monounsaturated lipids (g)** |
|  |
| **Polyunsaturated lipids (g)** |
|  |

| **Simple sugars (g)** |
| --- |
|  |
| **Alcohol (g)** |
|  |
| **Fibers (g)** |
|  |

| **Weight (kg)** |
| --- |
|  |
| **BMI (kg/m²)** |
|  |
| **Waist size (cm)** |
|  |
